# Talaromyces marneffei

**DOI:** 10.3201/eid2709.210318

**Published:** 2021-09

**Authors:** Monika Mahajan

**Affiliations:** Postgraduate Institute of Medical Education and Research, Chandigarh, India

**Keywords:** Talaromyces marneffei, Penicillium marneffei, fungi, fungal infections, penicilliosis, dimorphic fungus, talaromycosis, bamboo rats, Rhizomys sinensis, gymnothecium, zoonoses, Hubert Marneffe, Gabriel Segretain

## ***Talaromyces marneffei*** [t læ′ ɹɒ maɪ̯s ɪz mɑ:neɪ′]

**Figure 1:**
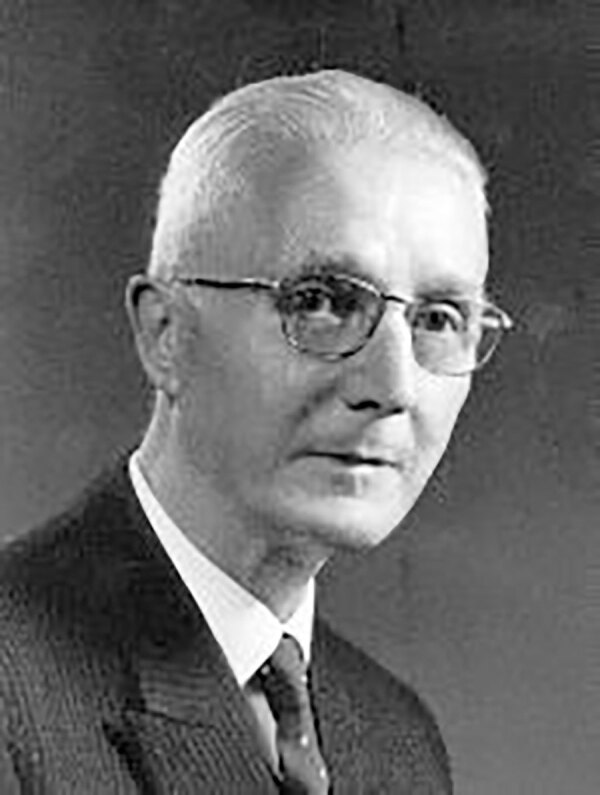
Hubert Marneffe (1901‒1970) Source: Wikimanche, Institut Pasteur, public domain.

*Talaromyces marneffei* (formerly *Penicillium marneffei*) is a thermally dimorphic fungus that causes talaromycosis, which was previously called penicilliosis. The genus name *Talaromyces* is derived from the Greek words *tálaros* (basket) and *múkēs* (mushroom). Talaros aptly describes the ascocarp known as a gymnothecium (composed of fine woven hyphae) in which asci are formed. Asexual stages of *Talaromyces* species were previously known as the species *Penicillium* of the subgenus *Biverticillium*. Capponi and Sureau isolated the fungus at Institute Pasteur de Dalat in Vietnam in 1955 from Chinese bamboo rats (*Rhizomys sinensis*). In 1959, Gabriel Segretain, after an accidental finger prick with a needle containing the yeast cells, described the fungus as a new species, naming it *Penicillium marneffei* in honor of Hubert Marneffe (1901‒1970), the Director of the Institute in Indochina (Figure 1).

**Figure 2:**
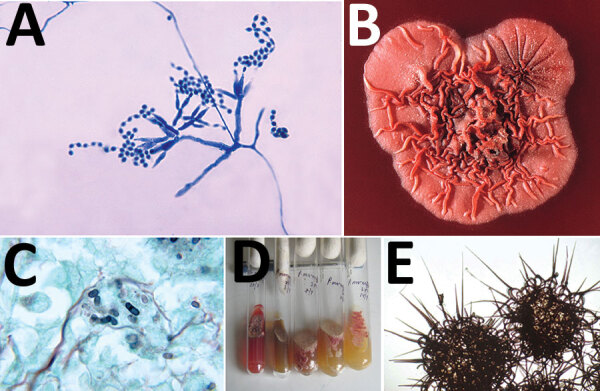
A) Ultrastructural morphology of *Talaromyces marneffei*, including chains of single-celled, teardrop-shaped conidia, each originating from its respective, flask-shaped phialide. Source: Libero Ajello, Centers for Disease Control and Prevention (https://phil.cdc.gov/Details.aspx?pid = 4240). B) Superior (front) view of a petri dish culture plate on which a wrinkled colony of *Penicillium marneffei* has been cultivated. Source: James Gathany, Centers for Disease Control and Prevention (https://phil.cdc.gov/Details.aspx?pid = 1879). C) Mouse testicle tissue specimen showing globe-shaped yeast cells of *T. marneffei* undergoing multiplication by binary fission not by mitosis (methenamine silver stain). Source: Libero Ajello, Centers for Disease Control and Prevention (https://phil.cdc.gov/Details.aspx?pid = 4235); D) Gradual conversion of mycelial phase of *T. marneffei* (growth at 25°C) to yeast phase on brain heart infusion agar after incubation at 37°C. Mycelial phase (first tube marked 25°C) shows diffusible red pigment. Source: Monika Mahajan, Postgraduate Institute of Medical Education and Research, Chandigarh, India; E) Loose network of hyphae of *T. marneffei* forming gymnothecium that contains asci. Source: https://istudy.pk/ascomycota-fruit-bodies/.

Talaromycosis affects persons who live in or visit Southeast Asia, southern China, or northeastern India, and are immunocompromised because of HIV/AIDS, cancer, organ transplant, or adult-onset immunodeficiency syndrome (Figure 2). This disease occurs after inhalation of aerosolized fungal spores from the environment. Although the precise reservoir is unknown, *T. marneffei* is found in bamboo rats.

## References

[1] Pitt JI. *Penicillium* and *Talaromyces*. In: Batt C. Patel P, editors. Encyclopedia of food microbiology. New York: Elsevier; 2014. p. 6–13.

[2] Talaromycosis (formerly penicilliosis) [cited 2021 Jun 10]. https://www.cdc.gov/fungal/diseases/other/talaromycosis.html

[3] Tsang C-C, Lau SKP, Woo PCY. Sixty years from Segretain’s description: what have we learned and should learn about the basic mycology of *Talaromyces marneffei?* Mycopathologia. 2019;184:721–9. 10.1007/s11046-019-00395-y 31599369

[4] Vanittanakom N, Cooper CR Jr, Fisher MC, Sirisanthana T. *Penicillium marneffei* infection and recent advances in the epidemiology and molecular biology aspects. Clin Microbiol Rev. 2006;19:95–110. 10.1128/CMR.19.1.95-110.2006 16418525 PMC1360277

